# Supplementary material for: Correlated states controlled by a tunable van Hove singularity in moiré WSe2 bilayers
Source: Nat Commun. 2025 Feb 25;16:1959. doi: 10.1038/s41467-025-57235-5 (PMC11861663; doi:10.1038/s41467-025-57235-5)
Supplement: Supplementary file 1 — Supplementary Information [file 41467_2025_57235_MOESM1_ESM.pdf]

Supplementary Information

Correlated states controlled by a tunable van Hove singularity in moiré WSe2 bilayers

Patrick Knüppel [1*], Jiacheng Zhu[1*], Yiyu Xia[1], Zhengchao Xia[1], Zhongdong Han[1], Yihang Zeng[1], Kenji Watanabe[2], Takashi Taniguchi[2], Jie Shan[1,3,4**], Kin Fai Mak[1,3,4**]

[1]Laboratory of Atomic and Solid-State Physics and School of Applied and Engineering Physics, Cornell University, Ithaca, NY, USA
[2]National Institute for Materials Science, 1-1 Namiki, 305-0044 Tsukuba, Japan
[3]Kavli Institute at Cornell for Nanoscale Science, Ithaca, NY, USA
[4]Max Planck Institute for the Structure and Dynamics of Matter, Hamburg, Germany

*These authors contributed equally
**Corresponding authors. Email: jie.shan@cornell.edu; kinfai.mak@cornell.edu

Supplementary Figures

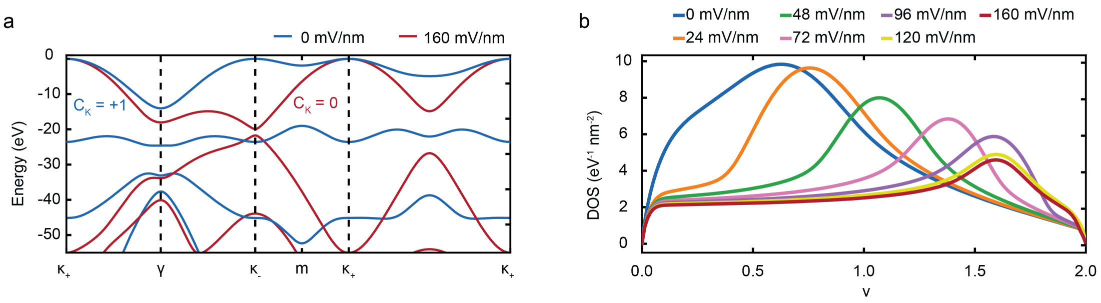

Supplementary Figure 1 | Electric-field tuned moiré band structure. a, Continuum model band structure of 2.7-degree twisted WSe_2 under electric field $E = 0$ (blue) and 160 mV/nm (red). The topmost moiré valence bands of the K-valley state are shown. The bands are more dispersive under a finite electric field. The first moiré band has Chern number 1 and 0 for E = 0 and 160 mV/nm, respectively. **b,** Filling factor ν dependence of the electronic density of states (DOS) for different electric fields E. As E increases, the van Hove singularity disperses to higher filling factors and the corresponding DOS maximum decreases

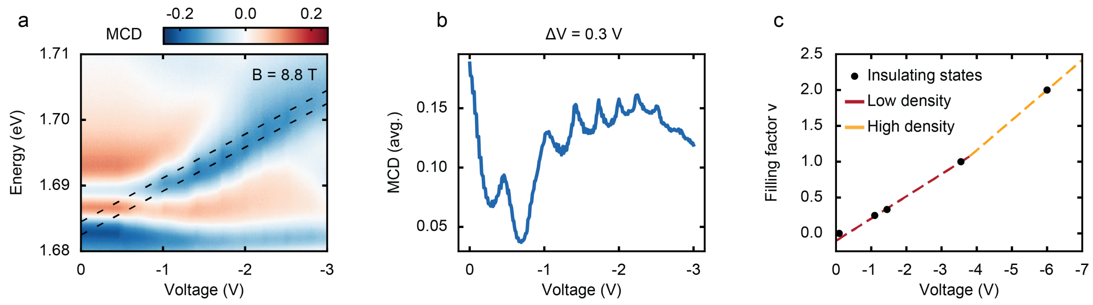

Supplementary Figure 2 | Twist angle calibration. a, Magnetic circular dichroism (MCD) spectrum of the intralayer exciton for 2.7-degree twisted WSe_2 as a function of (symmetric) gate voltage under 8.8 T at 1.6 K. The gate voltage varies doping density but not electric field in the sample. The periodic modulations of MCD show the formation of Landau levels (LLs). **b,** Gate voltage dependence of spectrally averaged MCD over the window between the dashed lines in **a**. The LL spacing is determined to be $\Delta V = 0.3$ V. **c,** Filling factor of the insulating states (identified from 2s sensing) versus gate voltage. A two-piece linear interpolation is used to convert the voltage to filling factor and to determine the moiré density and twist angle (Methods).

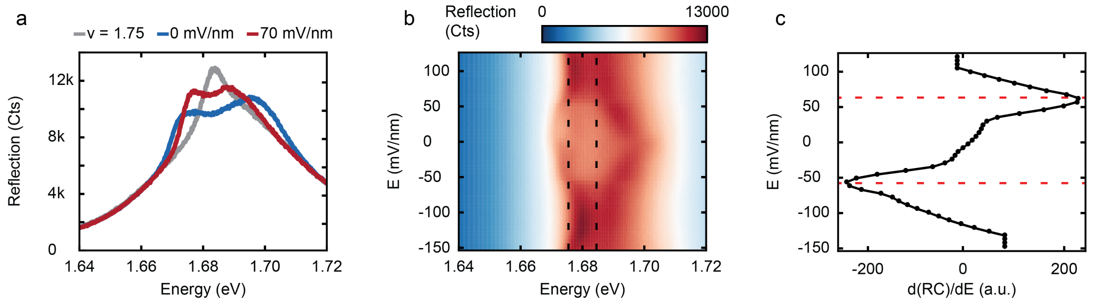

Supplementary Figure 3 | Determination of the layer-hybridized and layer-polarized phase boundary. a, Raw optical reflection spectra of intralayer exciton at (ν, E) = (1, 0) (blue), (1, 70 mV/nm) (red), and (1.7, 0) (grey). **b,** Raw optical reflection spectrum of intralayer exciton at $\nu = 1$ as a function of electric field. The dashed lines denote the spectral window employed for integration. **c,** Electric-field derivative of the integrated reflection contrast (RC, horizontal axis) versus electric field E (vertical axis). The extrema denoted by the dashed lines define the phase boundary between the layer-hybridized and layer-polarized regions. All results are shown for 2.7-degree twisted WSe_2 at 1.6 K.

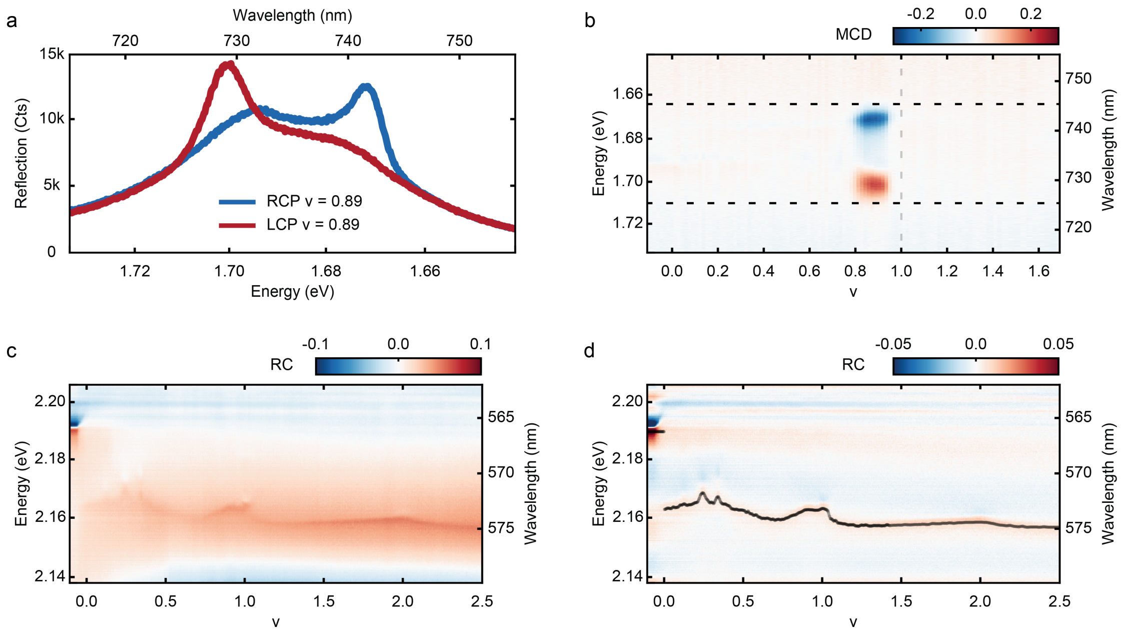

Supplementary Figure 4 | Analysis of optical reflection spectra. a, Raw optical reflection spectrum of intralayer exciton for the right and left circularly polarized light (filling factor $\nu = 0.89$, electric field $E = 0$ and magnetic field $B = 0$). **b,** Magnetic circular dichroism (MCD) spectrum as a function of filling factor ($E = 0$ and $B = 0$). The spectrally averaged MCD is obtained by integrating the absolute value of MCD over the spectral window between the horizontal dashed lines. The vertical dashed line marks $\nu = 1$. **c,** Filling dependence of reflection contrast (RC) spectrum of the sensor 2s exciton ($E = 0$ and $B = 0$). **d,** Result in **c** after removing a smooth background (Methods) to emphasize the 2s exciton resonance (black line). A spectral window of 2 nm around the 2s resonance is used to obtain the spectrally integrated reflection contrast of the sensor. All results are shown for 2.7-degree twisted WSe_2 at 1.6 K.

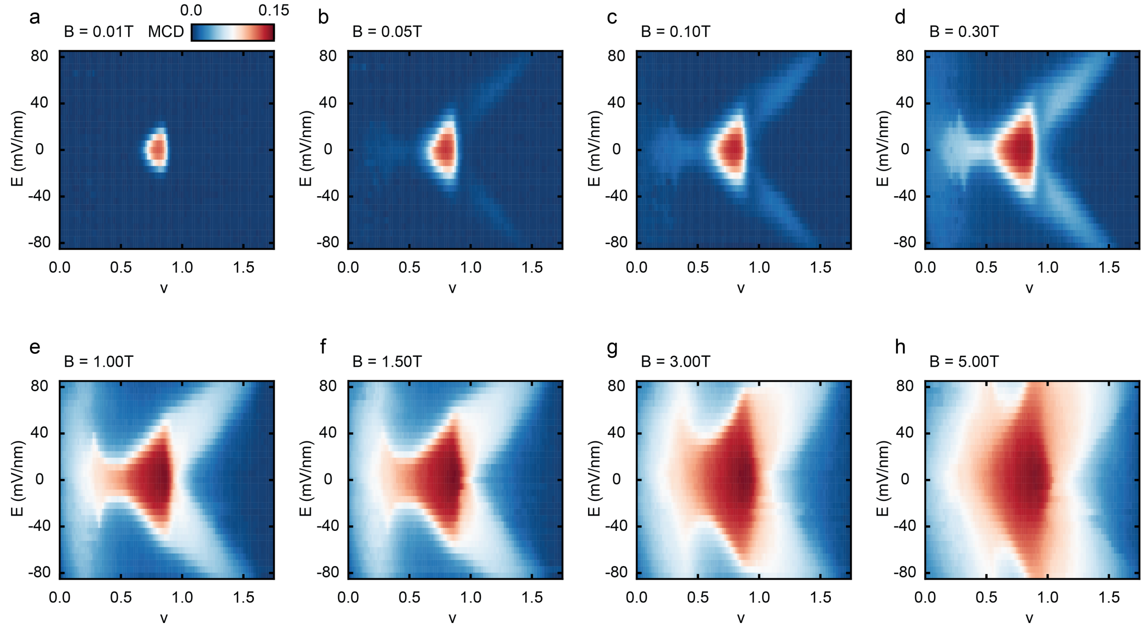

Supplementary Figure 5 | Magnetic circular dichroism (MCD) maps. a-h, MCD as a function of filling factor ν and electric field E under magnetic field $B = 0$ T (**a**), 0.05 T (**b**), 0.1 T (**c**), 0.3 T (**d**), 1.0 T (**e**), 1.5 T (**f**), 3.0 T (**g**) and 5.0 T (**h**). All results are shown for 2.7-degree twisted WSe_2 at 1.6 K.

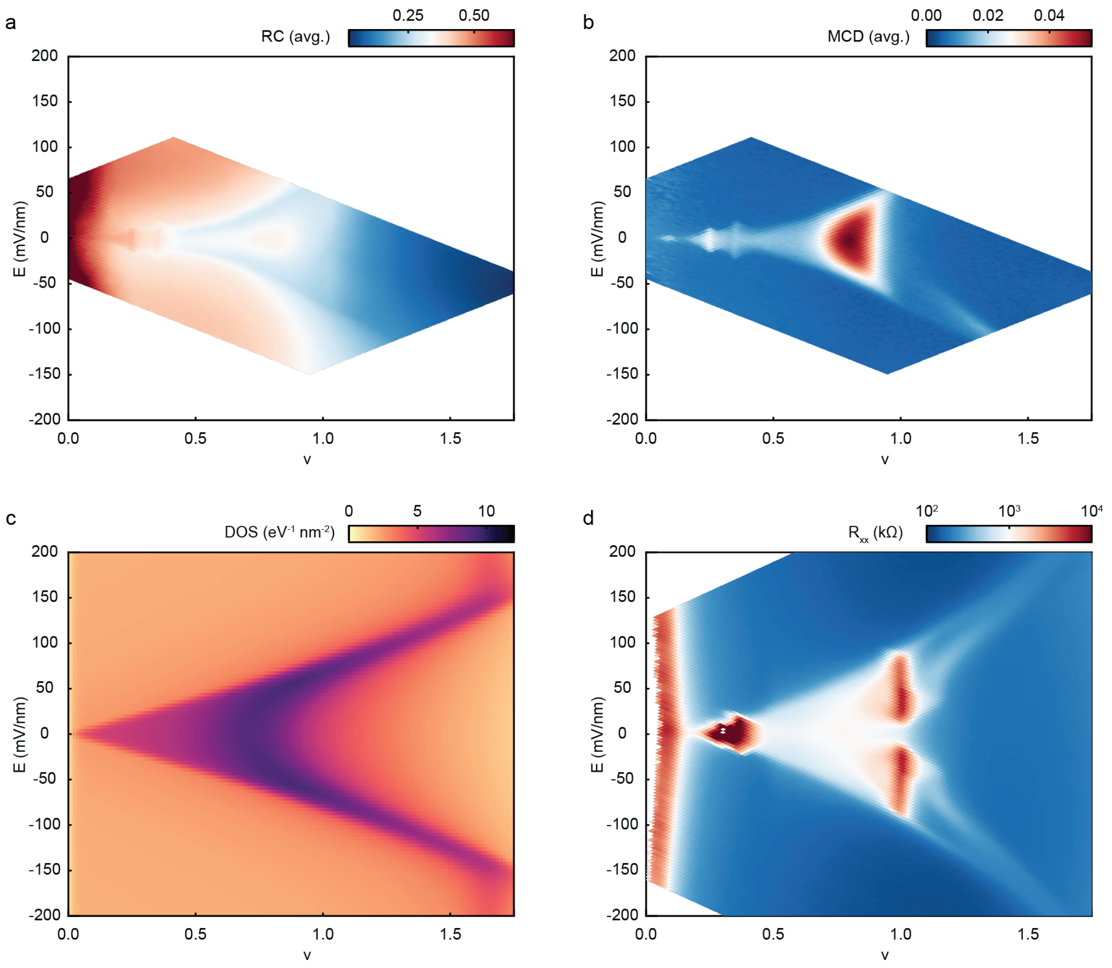

Supplementary Figure 6 | Comparison of electrical and optical measurements for 3.5-degree $tWSe_2$. a,b, Spectrally averaged optical reflection contrast (RC) for intralayer exciton (**a**) and magnetic circular dichroism (MCD) (**b**) as a function of filling factor ν and electric field E at 1.6 K. **c,** Calculated density of states (DOS) as a function of ν and E showing the evolution of the van Hove singularity (vHS) with E. **d,** The longitudinal resistance R_{xx} as a function of ν and E at 1.6 K. All measurements were taken under magnetic field $B = 0.1$ T. The vHS is manifested in **d** as a local resistance maximum. Its location in the phase diagram closely matches that for the enhanced MCD, supporting the picture of a vHS-enhanced magnetic response. The ferromagnetic metal phase is not observed in the 3.5-degree sample due to the weaker correlation effect.

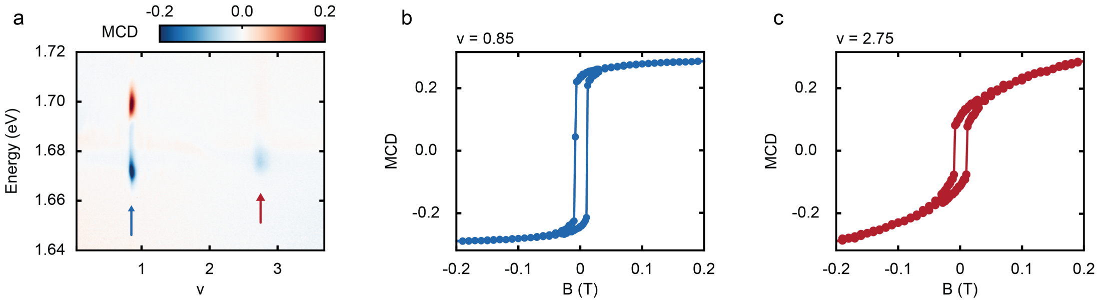

Supplementary Figure 7 | Stoner ferromagnetism in 2.5-degree twisted WSe_2. a, Spontaneous magnetic circular dichroism (MCD) spectrum as a function of filling factor ν. **b,c,** Magnetic-field (B) dependence of MCD at filling ν = 0.85 (**b**) and ν = 2.75 (**c**) denoted by the blue and red arrows in **a**, respectively. All results are shown for 1.6 K and zero electric field.

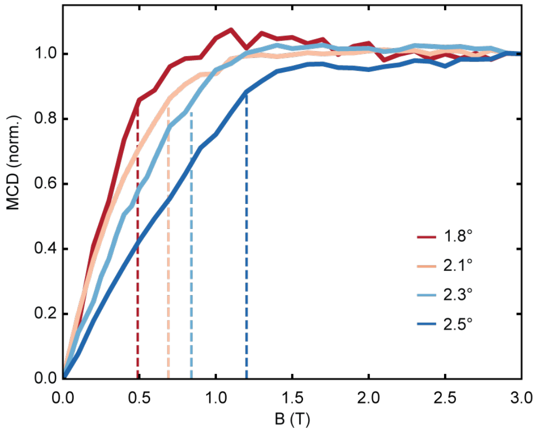

Supplementary Figure 8 | Determination of the saturation magnetic field. Magnetic-field dependence of the spectrally averaged magnetic circular dichroism (MCD) for filling factor ν = 1 and different twist angles. Dashed lines indicate the saturation field, which is defined as the field where MCD reaches 85% of its saturated value at high fields.

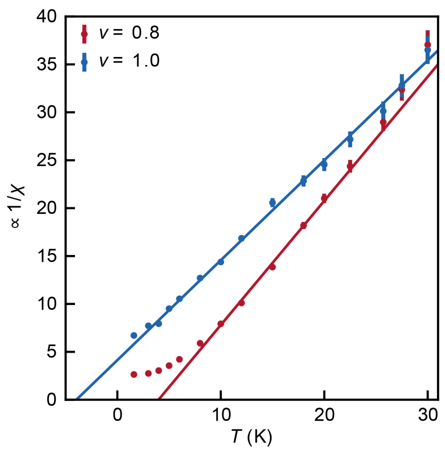

Supplementary Figure 9 | Temperature dependence of the magnetic susceptibility. The inverse magnetic susceptibility (extracted from the small-field MCD) can be described by the Curie-Weiss law (solid line) above 10 K with the best fit parameter for the Curie-Weiss temperature of $-(4.0 \pm 0.6)$ K for $\nu = 1$ and 4.0 ± 0.2 for $\nu = 0.8$ (see Methods).
